# Predictors of ICU Admission in Children with COVID-19: Analysis of a Large Mexican Population Dataset

**DOI:** 10.3390/jcm12103593

**Published:** 2023-05-22

**Authors:** Martha I. Cárdenas-Rojas, José Guzmán-Esquivel, Efrén Murillo-Zamora

**Affiliations:** Unidad de Investigación en Epidemiología Clínica, Instituto Mexicano del Seguro Social, Av. Lapislázuli 250, Col. El Haya, Villa de Álvarez 28984, Mexico

**Keywords:** child, COVID-19, pandemics, respiratory insufficiency, intensive care units

## Abstract

Children, although mostly affected mildly or asymptomatically, have also developed severe coronavirus disease 2019 (COVID-19). This study aims to assess potential predictors of intensive care unit (ICU) admission in a large population (*n* = 21,121) of children aged 0–9 years with laboratory-confirmed disease. We performed a cross-sectional analysis of a publicly available dataset derived from the normative epidemiological surveillance of COVID-19 in Mexico. The primary binary outcome of interest was admission to the ICU due to respiratory failure. Results showed that immunosuppressed children and those with a personal history of cardiovascular disease had a higher likelihood of being admitted to the ICU, while increasing age and the pandemic duration were associated with a lower likelihood of admission. The study’s results have the potential to inform clinical decision-making and enhance management and outcomes for children affected by COVID-19 in Mexico.

## 1. Introduction

Coronavirus disease 2019 (COVID-19) is a highly infectious disease caused by the severe acute respiratory syndrome coronavirus 2 (SARS-CoV-2) virus that has affected millions of people worldwide. The COVID-19 pandemic has caused a significant disease burden in Mexico since the first case was confirmed on 27 February 2020. As of 10 April 2023, the country has reported more than 7.5 million cases and nearly 335 thousand deaths [1].

The disease has resulted in a major strain on the Mexican healthcare system. Additionally, the COVID-19 pandemic has exacerbated pre-existing health disparities in Mexico, with vulnerable populations such as indigenous communities, low-income individuals, and those with underlying health conditions being disproportionately affected [2,3].

While the disease has primarily affected adults, children have also been affected. Although most COVID-19 cases in children are mild or asymptomatic, some children develop severe forms of the disease, leading to hospitalization and even death [4]. For a discrete-time epidemic model that accounts for disease severity and includes both asymptomatic and symptomatic carriers, one can refer to Ripoll J’s work [5], where model parameters can be fitted to available epidemiological data.

The identification of predictors of COVID-19 severity in children is essential for improving clinical management and outcomes in this vulnerable population [6]. Several studies have evaluated predictors of severe COVID-19 in adults, including age, comorbidities, and biomarkers such as D-dimer and C-reactive protein [7]. However, less is known about the predictors of severe COVID-19 in children.

In this study, our aim was to assess potential predictors of intensive care unit (ICU) admission in a large population of children aged 9 years and younger with laboratory-confirmed SARS-CoV-2 infection. The results of this study have the potential to inform clinical decision-making and enhance management and outcomes for children affected by COVID-19.

## 2. Materials and Methods

We performed a cross-sectional analysis of a publicly available dataset derived from the normative epidemiological surveillance of COVID-19 in Mexico (accessed from https://www.gob.mx/salud/documentos/datos-abiertos-bases-historicas-direccion-general-de-epidemiologia on 6 April 2023). The dataset was made public by the General Directorate of Epidemiology, and is web-based. It encompasses all COVID-19 cases (both suspected and confirmed) that have been registered by public and private healthcare facilities since the start of the pandemic in the country.

We included patients aged 0 to 9 years at the time of symptom onset who tested positive for COVID-19 via nasal swabbing and reverse-transcription polymerase chain reaction (RT-PCR) or rapid antigenic testing. Patients with incomplete data were excluded from the analysis. Cases that occurred between 28 February 2020 and 31 March 2023, and met the eligibility criteria, were included in the analysis.

Medical and death certificates, if applicable, serve as the primary sources of data for our surveillance system. Demographic characteristics, along with other relevant clinical and epidemiological data, were routinely collected and included in our analysis. The primary binary outcome of interest in this study was admission to the intensive care unit (ICU) due to respiratory failure, which was defined as the need for mechanical ventilatory support and an endotracheal tube.

In addition, we examined the impact of the COVID-19 pandemic progression on the risk of ICU admission. We defined this as the time (in weeks) from the onset of the pandemic in Mexico (27 February 2020) to the date of symptom onset. All analyzed exposures were obtained from the previously mentioned surveillance system.

Summary statistics were calculated. We used unconditional logistic regression models to identify factors that predict admission to the ICU and we computed odds ratios (ORs) with 95% confidence intervals. Ambulatory and hospitalized patients who did not require admission to the ICU were categorized as negative for the outcome of interest. Cohen’s d statistic was used to assess the effect size of the obtained estimators.

As we analyzed fully anonymized and publicly available data, the need for review and approval from an ethics committee was waived.

## 3. Results

Data from 21,121 children were analyzed. The majority of the participants were male (53%, n = 11,184/21,121), and the mean age was 5.2 ± 2.9 years. At the time of our analysis, the most recently enrolled patient had a symptom onset of 9 July 2022, as per the audited database.

A total of 2463/21,121 children (11.7%) required non-ambulatory medical management, and among them 364/2463 (14.8%) were admitted to the intensive care unit due to respiratory failure. About one out of five children admitted to the ICU had a fatal outcome (20%, n = 73/364).

The characteristics of the enrolled children for selected variables, as well as their ICU admission status, are summarized in Table 1. When compared to patients who did not require ICU admission, those who received medical management in an ICU were younger and experienced their cases earlier in the pandemic. Among the comorbid conditions analyzed, immunosuppression (of any cause) and cardiovascular disease (CVD, of any kind) were more frequent in children who required ICU admission.

The multiple regression model presented in Table 2 shows that immunosuppressed children (OR = 4.76, 95% CI 2.97–7.63) and those with a personal history of CVD (OR = 5.04, 95% CI 2.87–8.86) were more likely to be admitted to the ICU. In contrast, increasing age (compared to children under 1 year old: 1–4 years old, OR = 0.12, 95% CI 0.10–0.16; 5–9 years old, OR = 0.04, 95% CI 0.03–0.05) and the time elapsed from the pandemic onset to symptom onset (for each additional week: OR = 0.98, 95% CI 0.97–0.99) were associated with a lower likelihood of ICU admission. All d statistics for the model and each evaluated predictor were below 0.06, indicating that the effect sizes could be interpreted as small.

## 4. Discussion

Our findings indicate that immunosuppressed children and those with a personal history of cardiovascular disease are at a higher risk of ICU admission due to COVID-19. It is crucial for healthcare providers to identify these high-risk groups and provide appropriate care to prevent severe outcomes. In contrast, older age and pandemic progression were associated with a lower risk of ICU admission. Nonetheless, we acknowledge that this study is limited by its retrospective design and reliance on primary sources of data.

Immunosuppression has been identified as a potential risk factor for severe COVID-19 disease [8]. Our analysis of 21,121 pediatric cases found that immunosuppressed children had 4.76-fold increased odds of admission to the intensive care unit due to respiratory failure compared to non-immunosuppressed children. This finding is consistent with previous studies in adult populations, which have demonstrated that immunosuppression is associated with a higher risk of severe COVID-19 disease and mortality [9].

The exact mechanism by which immunosuppression increases the risk of severe COVID-19 disease is still not fully understood, but it may be related to impaired viral clearance, reduced immune response to the virus, or increased susceptibility to secondary infections [10,11]. Further research is needed to better understand the underlying mechanisms and to develop targeted interventions for this vulnerable population.

The presence of CVDs has been identified as a potential risk factor for severe COVID-19 illness [12]. In our study, children with a personal history of any of these diseases had five times the odds of being admitted to the ICU compared to those without CVDs.

Congenital heart diseases, including congenital arrhythmia-channelopathy (LQT) and cardiomyopathy, are significant causes of heart failure in young Mexican children [13]. The epidemiology of these conditions is complex and multifactorial, involving a combination of genetic, environmental, and developmental factors. Several studies have reported an increasing prevalence of congenital heart diseases in Mexico, with variations in the prevalence rates depending on the specific type of heart disease and the region of the country [14]. Genetic factors, such as consanguinity and a high prevalence of certain gene mutations, may contribute to the increased prevalence of congenital heart diseases in the Mexican population [15]. Environmental factors, including maternal use of medications during pregnancy, exposure to infections, and poor prenatal care, may also play a role [16].

The pathophysiology of COVID-19 involves the binding of the virus to angiotensin-converting enzyme 2 (ACE2) receptors, which are widely expressed in the cardiovascular system [17]. Patients with preexisting CVDs, such as hypertension, coronary artery disease, and heart failure, may have upregulated ACE2 receptors, which could increase viral load and disease severity [18]. Furthermore, COVID-19 can exacerbate preexisting CVDs, leading to cardiovascular complications such as myocarditis, acute myocardial infarction, and arrhythmias [19]. Therefore, it is crucial to identify and closely monitor patients with preexisting CVDs who contract COVID-19 to improve their clinical outcomes.

The impact of gender on COVID-19 progression has been a topic of interest since the beginning of the pandemic. Studies have consistently shown that males are at a higher risk of severe illness and death due to COVID-19 compared to females [20,21]. It has been suggested that estrogen, which is present in higher levels in females, may play a protective role in COVID-19 by reducing inflammation and enhancing the immune response [22]. In our study sample of prepubertal children, we did not observe a significant difference in the risk of ICU admission between males and females. However, more research is needed to fully understand the mechanisms behind this gender difference and to identify potential interventions to improve outcomes for both males and females affected by COVID-19.

In our study, a personal history of type 1 diabetes mellitus was not associated with an increased risk for ICU admission. This finding is consistent with previously published studies [23].

We documented that pandemic evolution was associated with a decreased risk of ICU admission, which may reflect an improved understanding and management of the disease over time [24]. As the COVID-19 pandemic continues to spread globally, other factors that determine the observed scenario may include changes in the virulence of the virus, and differences in host susceptibility and immune response [25,26].

Furthermore, advances in COVID-19 vaccines have contributed to improved clinical outcomes and reduced mortality rates in COVID-19 patients [27]. To fully understand the complex interplay of these factors and develop effective strategies for preventing and treating severe COVID-19 illness over the course of the ongoing pandemic, further research is needed. In Mexico, COVID-19 vaccination in children began in October 2021, initially limited to chronically ill pediatric patients [28]. Vaccination efforts for children in the general population did not occur until mid-2022. Unfortunately, the analyzed dataset does not include information regarding the vaccination status.

## 5. Conclusions

Our findings suggest that immunosuppressed children and those with a personal history of cardiovascular disease are particularly vulnerable to severe disease and more likely to be admitted to the ICU. Increasing age was associated with a decreased risk of ICU admission, which may reflect age-related differences in immune response. The pandemic evolution was also found to be a factor in ICU admission, with each additional week associated with a slightly lower risk.

These findings underscore the need for continued efforts to mitigate the impact of COVID-19 on children, particularly those with underlying health conditions. Further research is needed to better understand the mechanisms underlying the increased risk of severe disease in vulnerable populations and to identify effective prevention and treatment strategies.

## Figures and Tables

**Table 1:** Characteristics of the study sample for selected variables, Mexico 2020–2023.

Characteristic	ICU Admission		*p*
	**No (*n* = 20,757)**	**Yes (*n* = 364)**	
Gender			
Female	9766 (47.1)	171 (47.0)	0.978
Male	10,991 (52.9)	193 (53.0)	
Age (median), years	6 (3–8)	0 (0–1)	<0.001
Age group, years			
<1	928 (4.5)	210 (57.7)	<0.001
1–4	7305 (35.2)	106 (29.1)	
5–9	12,524 (60.3)	48 (13.2)	
Time elapsed from pandemic start to symptoms onset (median), weeks	105 (53–106)	25 (22–53)	<0.001
Indigenous, self-perceived			
No	20,632 (99.4)	358 (98.4)	0.012
Yes	125 (0.6)	6 (1.6)	
Type 1 diabetes mellitus			
No	20,668 (99.6)	361 (99.2)	0.256
Yes	89 (0.4)	3 (0.8)	
COPD			
No	20,740 (99.9)	363 (99.7)	0.211
Yes	17 (0.1)	1 (0.3)	
Asthma			
No	20,375 (98.2)	362 (99.5)	0.068
Yes	382 (1.8)	2 (0.5)	
Immunosuppression (of any cause)			
No	20,545 (99.0)	335 (92.0)	<0.001
Yes	212 (1.0)	29 (8.0)	
CVD (of any cause)			
No	20,637 (99.4)	344 (94.5)	<0.001
Yes	120 (0.6)	20 (5.5)	
Obesity			
No	20,509 (98.8)	359 (98.6)	0.756
Yes	248 (1.2)	5 (1.4)	
Chronic kidney disease			
No	20,719 (99.8)	363 (99.7)	0.686
Yes	38 (0.2)	1 (0.3)	

Abbreviations: ICU, Intensive Care Unit; COPD, Chronic Pulmonary Obstructive Disease; CVD, Cardiovascular disease. Notes: (1) Absolute frequencies (*n*) and relative frequencies (%) are presented, except when the median is specified, in which case the interquartile range is also provided. (2) The p-value obtained from chi-squared or U test is reported accordingly. (3) The data from 2023 only include patients from the first trimester of the year.

**Table 2:** Predictors of ICU admission, Mexico 2020–2023.

Characteristic	OR (95% CI), *p*	
	Bivariate Analysis	Multiple Analysis
Gender		
Female	1.00	1.00
Male	1.01 (0.81–1.23), 0.978	1.01 (0.81–1.27), 0.913
Age group (years)		
<1	1.00	1.00
1–4	0.06 (0.05–0.08), <0.001	0.12 (0.10–0.16), <0.001
5–9	0.02 (0.01–0.03), <0.001	0.04 (0.03–0.05), <0.001
Time elapsed from pandemic start to symptoms onset (weeks)	0.965 (0.961–0.969), <0.001	0.979 (0.975–9.983), <0.001
Asthma		
No	1.00	1.00
Yes	0.29 (0.07–1.19), 0.086	0.24 (0.05–1.14), 0.072
Immunosuppression (of any cause)		
No	1.00	1.00
Yes	8.39 (5.61–12.55), <0.001	4.76 (2.97–7.63), <0.001
CVD (of any cause)		
No	1.00	1.00
Yes	10.00 (6.15–16.24), <0.001	5.04 (2.87–8.86), <0.001

Abbreviations: ICU, Intensive Care Unit; OR, Odds Ratio; CI, Confidence Interval; CVD, Cardiovascular disease. Notes: (1) OR and 95% CI from the multiple model were adjusted by the variables presented in the table; (2) the data from 2023 only include patients from the first trimester of the year.

## Data Availability

Publicly available datasets were analyzed in this study. These data can be found here: https://www.gob.mx/salud/documentos/datos-abiertos-bases-historicas-direccion-general-de-epidemiologia (accessed on 6 April 2023).
